# Indocyanine Green and Radioisotope Tracers for Sentinel Lymph Node Mapping in Early-Stage Cervical Cancer: A Prospective Comparative Study

**DOI:** 10.7759/cureus.82644

**Published:** 2025-04-20

**Authors:** Shinichi Togami, Nozomi Fufuzono, Mika Fukuda, Hiroaki Kobayashi

**Affiliations:** 1 Department of Obstetrics and Gynecology, Faculty of Medicine, Kagoshima University, Kagoshima, JPN

**Keywords:** endometrial cancer, indocyanine green, near-infrared fluorescent imaging, radio-isotope, sentinel lymph node

## Abstract

Aim

The aim of this study is to compare the number and pattern of sentinel lymph nodes (SLNs) identified by indocyanine green (ICG) and radioisotope (RI) tracers in early-stage cervical cancer (CC) and evaluate the clinical implications of multiple SLN detections by ICG.

Methods

This prospective study included 88 patients with histologically confirmed CC who underwent semi-radical/radical hysterectomy or trachelectomy with SLN mapping using both ICG and RI tracers at a single tertiary center between April 2017 and December 2024. ICG was injected into the cervix immediately before surgery, and RI (99m technetium-phytate) was administered the day before surgery. SLNs were detected intraoperatively using near-infrared fluorescence and gamma-ray probes. SLN detection counts were compared bilaterally between the two tracers.

Results

The bilateral SLN detection rate was equivalent for both tracers (92.0%). However, ICG identified significantly more SLNs per patient than RI (mean: 2.56 vs. 2.10; *p* = 0.028), with more frequent multiple SLN detection on both pelvic sides (right, 27% vs. 12.3%; left, 32.8% vs. 11.5%; *p* < 0.0001). A moderate correlation was observed between SLN counts obtained using the two methods (r = 0.535).

Conclusion

ICG is as effective as RI in bilateral SLN detection and more sensitive in detecting multiple nodes. This likely reflects ICG’s higher sensitivity owing to its lower molecular weight and deeper lymphatic penetration. However, the risk of SLN over-identification should be considered during clinical interpretation and surgical planning.

## Introduction

Lymph node metastasis is a critical prognostic factor in cervical cancer (CC), directly influencing survival outcomes and the selection of adjuvant therapy [1,2]. Therefore, accurate nodal assessment is essential, particularly in early-stage disease, to guide optimal treatment strategies. Traditionally, pelvic lymphadenectomy has been the standard method for lymph node evaluation; however, it is associated with potential complications such as lymphedema, nerve injury, and lymphocele formation [3,4].

Sentinel lymph node (SLN) biopsy has emerged as a less invasive alternative that enables targeted lymphatic mapping by identifying the first lymph node draining from the tumor site. Its clinical utility in early-stage CC has been validated in several prospective studies, most notably the SENTICOL I and II trials. These studies demonstrated the high sensitivity and negative predictive value of SLN biopsy, supporting its role in guiding individualized treatment planning [5,6]. Moreover, the SENTICOL II trial found no significant differences in disease-free or overall survival between patients who underwent SLN biopsy alone and those who underwent full pelvic lymphadenectomy, further supporting the oncological safety of this approach [7].

Historically, technetium-99m radiocolloid and blue dyes have been used for SLN mapping [8,9]. Indocyanine green (ICG) has recently been used as a fluorescent tracer with superior visualization capabilities in near-infrared imaging. ICG offers real-time lymphatic mapping without radiation exposure and has shown excellent compatibility with minimally invasive robot-assisted procedures. Consequently, ICG-based SLN mapping has been increasingly adopted in clinical practice [10,11].

However, some studies have suggested that ICG may identify multiple SLNs more frequently than radioisotope (RI), raising concerns about potential overdetection and its impact on diagnostic accuracy. Identifying excessive SLNs can lead to unnecessary dissection or compromise the specificity of the SLN biopsy. However, few studies have directly compared the number of SLNs detected using ICG and RI in the same patients.

This study aims to compare the number of SLNs identified using ICG and RI in patients with early-stage CC who underwent both techniques and to evaluate the clinical significance of detecting multiple SLNs with ICG.

## Materials and methods

This prospective observational study was conducted at Kagoshima University Hospital and included patients who underwent surgery for CC with a concurrent SLN biopsy between April 2017 and December 2024. The study protocol was approved by the Institutional Review Board (IRB) of Kagoshima University Hospital (No. 20-K04), and written informed consent was obtained from all participants. This prospective observational study was conducted as part of a registered clinical trial (jRCTs071200026) at Kagoshima University Hospital.

Eligibility criteria

Patients were included if they met the following criteria: histologically confirmed CC; underwent abdominal or minimally invasive surgery, including semi-radical/radical hysterectomy or semi-radical/radical trachelectomy; and received SLN mapping using a hybrid method that combined both RI and ICG techniques. Patients who underwent SLN mapping using only one tracer (either RI or ICG) were excluded from the analysis. Demographic and surgical data were extracted from electronic medical records.

SLN mapping procedure

SLN mapping was performed using a hybrid approach. For the RI method, 111 MBq of technetium-99m phytate was injected into the four quadrants of the cervix the day before surgery. Subsequently, lymphoscintigraphy and single-photon emission computed tomography/computed tomography (SPECT/CT) were performed to localize the SLNs.

For the ICG method, 1 mL of 10-fold diluted ICG was injected into two quadrants of the cervix immediately before surgery. During surgery, SLNs were identified using a near-infrared fluorescence imaging system and confirmed using a gamma probe to detect radioactive (hot) nodes. All fluorescent and hot nodes were excised.

Statistical analysis

Statistical analyses were conducted using the JMP Pro software (version 14.0; SAS Institute Inc., Cary, NC, USA). The number of SLNs identified using ICG and RI was compared on the right, left, and bilateral pelvic sides. Node counts detected by each method were summarized as means with standard deviations. Differences in SLN count distributions were assessed using the chi-square and Fisher’s exact tests. Additionally, the correlation between the ICG- and RI-detected node counts was computed using Pearson’s correlation coefficient, and a bivariate scatter plot was generated to visualize this association. Categorical variables were calculated using the chi-square test. A p-value < 0.05 was considered statistically significant.

## Results

Table 1 summarizes the clinicopathological characteristics of the 88 patients included in this study. The mean age and body mass index (BMI) were 41 years and 22.2 kg/m², respectively. The predominant histological subtypes were SCC and adenocarcinoma, accounting for 59 (67%) and 25 (28%) patients, respectively. Laparoscopic radical or semi-radical hysterectomy was the most performed surgical procedure, conducted in 50 patients (57%). Based on the 2018 International Federation of Gynecology and Obstetrics (FIGO) classification, stage IB1 was the most frequent stage, observed in 55 patients (63%). Lymphovascular space invasion was identified in 33 patients (37%), and cervical stromal invasion in 68 patients (78%).

**Table 1:** Clinicopathological characteristics BMI, body mass index; SCC, squamous cell carcinoma; FIGO, International Federation of Gynecology and Obstetrics; LVSI, lymphovascular space invasion

Characteristics	Patients (N = 88)
Median age (years)	41 (28–78)
Median BMI (kg/m^2^)	22.2 (16.5–50.4)
Final pathology	
SCC	59 (67%)
Adenocarcinoma	25 (28%)
Adeno-SCC	2 (2%)
Other	2 (2%)
Surgical procedure	
Laparoscopic radical/semi-radical hysterectomy	50 (57%)
Radical/semi-radical hysterectomy	14 (16%)
Radical/semi-radical trachelectomy	13 (15%)
Robotic radical/semi-radical hysterectomy	7 (8%)
Robotic radical/semi-radical	4 (4%)
FIGO stage (2018)	
IA2	15 (17%)
IB1	55 (63%)
IB2	11 (12%)
IIA1	2 (2%)
IIIC1	5 (6%)
LVSI	
No	55 (63%)
Yes	33 (37%)
Cervical stromal invasion	
No	6 (7%)
<1/2	62 (71%)
1/2<	19 (22%)

Table 2 compares the pelvic SLN detection rates between the RI and ICG methods. There was statistical significance in bilateral detection rates between the ICG and RI methods (p = 0.1577). Both methods achieved high bilateral detection (92%), indicating comparable performance.

**Table 2:** Comparison of the pelvic SLN detection rate between RI and ICG (n = 88) ICG, indocyanine green; RI, radioisotope; SLN, sentinel lymph node

Pelvic SLN detection	RI (n, %)	ICG (n, %)	p-value (Chi-square)
Bilateral	81 (92.0%)	81 (92.0%)	-
Unilateral	7 (8.0%)	6 (6.8%)	
None	0 (0.0%)	1 (1.1%)	
Total	88 (100%)	88 (100%)	0.16

In our analysis of SLN detection in CC, the ICG method identified significantly more multiple SLNs than the RI method (p<0.0001) (Table 3). Although side-specific comparisons (right and left pelvic regions) did not reach statistical significance, the overall number of detected SLNs per case was significantly higher with ICG, suggesting potential for overdetection or higher sensitivity.

**Table 3:** Correlation and comparison of pelvic sentinel lymph nodes identified by ICG and RI methods (n = 88) ICG, indocyanine green; RI, radioisotope

Region	ICG Detection Pattern	RI Detection Pattern	Pearson χ^²^	p-value	Fisher’s Exact Test
Right pelvis	1 node vs. multiple/none	1 node vs. multiple/none	7.19	0.1261	NS
Left pelvis	1 node vs. multiple/none	1 node vs. multiple/none	3.90	0.4195	NS
Total nodes	1 vs. multiple nodes	1 vs. multiple nodes	35.66	< 0.0001	p < 0.0001

A statistically significant positive correlation was observed between the number of pelvic SLNs identified using ICG and RI (r = 0.535, p<0.0001) (Table 4). While both methods showed concordant trends, the ICG method yielded a higher mean number of SLNs (2.56 ± 1.13) compared with RI (2.10 ± 0.53), suggesting that ICG may lead to more extensive SLN mapping or potential over-identification.

**Table 4:** Comparison of the number of pelvic sentinel lymph nodes identified by ICG and RI methods (n = 88) ICG, indocyanine green; RI, radioisotope; SLN, sentinel lymph node

Variable	ICG	RI	Statistical Measure	Value	95% CI (Lower–Upper)	p-value
Mean number of SLNs	2.56	2.10	-	-	-	-
Standard deviation	± 1.13	± 0.53	-	-	-	-
Correlation coefficient (Pearson)	-	-	r	0.535	0.367 – 0.669	< 0.0001
Covariance	-	-	-	0.333	-	-

## Discussion

In this study, we compared the number of SLNs identified using the RI and ICG methods in patients undergoing SLN mapping for CC using a hybrid approach. While no significant difference in bilateral detection rates was observed between the two methods, the number of SLNs identified per patient was significantly higher with the ICG method. Notably, the proportion of patients with multiple SLNs was substantially higher in the ICG group compared with the RI group.

This finding likely reflects the high sensitivity of ICG as a tracer. ICG, with its low molecular weight (775 Da), rapidly flows through lymphatic channels, enabling it to reach more distal lymph nodes than colloidal tracers such as technetium-99m-labeled phytate. Consequently, ICG may detect primary or “true” SLN and secondary (second-echelon) nodes, potentially leading to overdetection.

Our results are consistent with our previous findings on endometrial cancer [12], where ICG identified a significantly higher number of SLNs compared with RI. This suggests that ICG’s tendency to identify multiple SLNs may be a common phenomenon across gynecologic malignancies.

Several studies have supported the utility of ICG for SLN mapping. Baeten et al. [13] reported that ICG showed a significantly higher bilateral detection rate compared with the combination of RI and blue dye (90.3% vs. 73.5%, respectively). Similarly, a meta-analysis by Wang et al. [14] demonstrated that ICG achieved the highest bilateral detection rate (85%) compared with RI plus dye (76%) and RI alone (63%). These findings highlight ICG’s superior performance regarding its detection capability and intraoperative real-time visualization.

However, recent studies have indicated that ICG alone may achieve SLN detection rates comparable to those of hybrid methods [15]. Buda et al. [16] found no significant difference in detection rates between ICG alone and RI + dye in robot-assisted CC surgery. The ongoing SENTICOL III trial [6] has also adopted ICG as the sole tracer, reflecting a growing trend toward eliminating RI from SLN protocols owing to its logistical burdens, radiation exposure, and limited availability in some regions.

Despite its advantages [13,14], ICG-based mapping has some limitations. The increased number of SLNs detected may prolong surgical time, increase the burden of pathological examination, and complicate the differentiation between true SLNs and second-echelon nodes. In CC, where lymphatic drainage pathways are highly variable and often involve the obturator, internal iliac, and parametrial nodes, accurate identification of the true SLN highly depends on the surgeon’s experience and anatomical knowledge.

This study has some limitations. First, the relatively small sample size may have limited the statistical power and generalizability of the findings. Second, as a single-institution study, the results may not be fully representative of broader clinical practice.

## Conclusions

ICG is a highly effective tracer for SLN mapping in the CC, offering superior sensitivity and high bilateral detection rates. However, its potential for overdetecting SLNs, possibly due to distal lymphatic migration, should be carefully considered. Future efforts should focus on developing anatomically informed SLN mapping protocols tailored to ICG’s properties to optimize diagnostic accuracy.
